# New Formulation of a Subunit Vaccine Candidate against *Lawsonia intracellularis* Increases Humoral and Cellular Immune Responses

**DOI:** 10.3390/vaccines11121817

**Published:** 2023-12-04

**Authors:** Santiago Salazar, María Francisca Starck, Milton F. Villegas, Jannel Acosta, Oliberto Sánchez, Eduardo Ramos, Estefanía Nova-Lamperti, Jorge R. Toledo, Paula Gädicke, Álvaro Ruiz, Alaín González, Raquel Montesino

**Affiliations:** 1 Biotechnology and Biopharmaceuticals Laboratory, Pathophysiology Department, School of Biological Sciences, Universidad de Concepción, Victor Lamas 1290, Concepción P.O. Box 160-C, Chile; sasalazar@udec.cl (S.S.); mstarck@udec.cl (M.F.S.); miltonfvillegas@udec.cl (M.F.V.); janacosta@udec.cl (J.A.); edramos@udec.cl (E.R.); jotoledo@udec.cl (J.R.T.); 2 Pharmacology Department, School of Biological Sciences, Universidad de Concepción, Victor Lamas 1290, Concepción P.O. Box 160-C, Chile; osanchez@udec.cl; 3 Molecular and Translational Immunology Laboratory, Clinical Biochemistry and Immunology Department, Pharmacy Faculty, Universidad de Concepción, Victor Lamas 1290, Concepción P.O. Box 160-C, Chile; enovalamperti@gmail.com; 4 Pathology and Preventive Medicine Department, School of Veterinary Sciences, Universidad de Concepción, Avenida Vicente Méndez 595, Chillan P.O. Box 537, Chile; pgadicke@udec.cl (P.G.); aruiz@udec.cl (Á.R.); 5 Faculty of Basic Sciences, University of Medellin, Cra. 87 No. 30-65, Medellin P.C. 050026, Antioquia, Colombia; agonzalezp@udemedellin.edu.co

**Keywords:** *Lawsonia intracellularis*, subunit vaccine, ISA 660 VG, pig immune response, water-in-oil formulation

## Abstract

Previously, we designed a subunit vaccine candidate based on three *L. intracellularis* antigens with promising results in pigs. In this study, antigens were produced individually to achieve an even antigen ratio in the formulation. The emulsion characterization included the drop size and the mechanical and thermal stability. Immune response was evaluated by indirect and sandwich ELISAs, qPCR, and flow cytometry. The vaccine candidate’s safety was assessed by histopathology and monitoring the clinical behavior of animals. The average production yielded for the chimeric antigen as inclusion bodies was around 75 mg/L. The formulation showed mechanical and thermal stability, with a ratio Hu/Ho > 0.85 and a drop size under 0.15 nm. Antigens formulated at a ratio of 1:1:1 induced a significant immune response in inoculated pigs that persisted until the end of the experiment (week 14). The dose of 200 μg significantly activated cellular response measured by transcriptional and translational levels of cytokines. The cell proliferation assay revealed an increment of lymphocytes T CD4^+^ at the same dose. Animals gained weight constantly and showed proper clinical behavior during immunization assays. This research demonstrated the immunological robustness of the new subunit vaccine candidate against Porcine Proliferative Enteropathy evenly formulated with three chimeric antigens of *L. intracellularis*.

## 1. Introduction

The intracellular pathogen *L. intracellularis* causes Porcine Proliferative Enteropathy (PPE), an enteric disease distributed all over the world that affects the swine industry [1]. As pork meat is the second most consumed worldwide https://www.oecd-ilibrary.org/agriculture-and-food/data/oecd-agriculture-statistics_agr-data-en, accessed on 4 July 2023, it is important to make efforts to solve this problematic situation. *L. intracellularis* multiplies in the apical region of enterocytes through a process called “energy parasitism”, allowing its survival against cellular oxidative stress by taking advantage of the host energy reserves with a complex enzymatic battery [2]. Intracellular bacterial replication is accompanied by the proliferation of immature intestinal crypts and villi cells [3], producing the thickened and corrugated appearance of the intestinal mucosa. These characteristic PPE macroscopic lesions are mainly generated in the jejunum and ileum. A few lesions are also observed in the cecum and colon [4,5,6]. At a microscopic level, crypt hyperplasia, the proliferation of immature enterocytes, and a decrease in the total or partial number of goblet cells are observed [7]. Clinical symptoms correlate with chronic and acute disease stages, ranging from mild weight loss to hemorrhagic diarrhea and death [8]. Undoubtedly, *L. intracellularis* causes significant economic losses in pig farming and constitutes, therefore, a veterinary health problem.

Campaigns to reduce *L. intracellularis* infections have mainly been based on the administration of antibiotics and vaccination. However, the current trend is to reduce antibiotics due to the negative impact on the environment, humans, and swine production. Some undesirable effects of antibiotics involve the generation of antibiotic-resistant bacteria that can be spread into the environment with highly zoonotic potential, the generation of similar antibiotic resistance mechanisms to those found in humans, and the damage of animal gastrointestinal microbiota by the excessive use of antibiotics [9,10]. Regarding vaccination, there are two commercial vaccines approved to prevent PPE. A live attenuated vaccine, Enterisol^®^ Ileitis (Boehringer Ingelheim Animal Health USA Inc., Duluth, GA, USA); and an inactivated one, Porcilis^®^ Ileitis (Merck Animal Health, Kenilworth, NJ, USA). Both vaccines have offered some protection degree to vaccinated animals [11,12,13,14,15,16,17]. However, the production of these kinds of vaccines requires bacterial replication in eukaryotic cell culture, which implies high production costs associated with the particularly challenging microorganism isolation and in vitro culture conditions [18,19]. Additionally, these vaccines do not allow for the proper serological differentiation of vaccinated and infected animals [20,21].

Recombinant subunit vaccines could overcome the limitations of currently approved vaccines against *L. intracellularis*. As these kinds of vaccines are only composed of one or a few proteins of the pathogen, virulence reversion is avoided when compared to a live attenuated vaccine, and vaccine manufacture becomes easier, faster, and cost-effective due to the disadvantages of *L. intracellularis* isolation and cultivation process mentioned before. Moreover, the enormous progress in the computational design of epitope-based vaccines could improve vaccine efficacy by selecting the accurate antigen composition, linkers and tags, and B and T epitopes, and such an improvement, in turn, facilitates antigen processing, increases the specificity of the vaccine candidate, and supports a long-lasting immune response [22,23]. A great breakthrough in the research of a recombinant vaccine against *L. intracellularis* was the sequencing of the bacterial genome [24,25], encouraging researchers to identify potential antigens against the pathogen to design and develop recombinant vaccine candidates [26,27,28]. In a previous study, our group produced a recombinant vaccine candidate to prevent PPE based on three antigenic proteins from *L. intracellularis* [29]. Bioinformatic software was used to analyze the bacterial genome and pick up the three proteins, considering the subcellular location, molecular weight, B and T epitopes, and antigenic potential previously described. An in silico structural analysis allowed for protein modification in order to improve their immunological characteristics. This multi-antigenic and chimeric vaccine candidate showed promising results by inducing an effective immune response and exhibiting protecting signs on immunized pigs upon challenge [29,30]. To make the vaccine candidate production and the downstream process feasible, DNA sequences coding chimeric proteins were cloned into the same plasmid to express the three antigens simultaneously in *E. coli* as inclusion bodies. This expression system allowed for a one-step antigen production, excluding laborious and time-consuming protein purification and renaturation stages. However, protein yield was not homogeneous for the three antigens because each protein had a differential expression pattern. Hence, the final formulation did not contain an equivalent concentration of the three antigens; therefore, the induction of a reproducible immune response and the generation of a consistent formulation of the vaccine candidate could be affected. To overcome this drawback, in this study, we evaluated the safety and the immune response induced by a new formulation of our vaccine candidate that comprises equal quantities of the three chimeric antigens.

## 2. Materials and Methods

### 2.1. Bacterial Strains and Animals

The Shuffle^®^ T7 *E. coli* strain (New England Biolabs, UK, Cat. No. C3029) produced the recombinant antigens. In vivo experiments complied with national guidelines and the authorization of the Ethical Committee of the University of Concepcion. Fifty-two healthy Duroc/Yorkshire piglets, four weeks old, acquired from an intensive pig’s production Chilean farm, with no history of clinical signs compatible with *L. intracellularis*, were used to determine the immune response of recombinant *L. intracellularis* antigens. The commercial Ileitis Antibody ELISA (Cat. No. 112986, SVANOVA, Uppsala, Sweden) was used to confirm that the pigs were free from *L. intracellularis*.

Six-week-old C57/BL6 female mice were acquired from the National Institute of Public Health (ISP, Santiago, Chile) for the emulsion stability evaluation. Animals were maintained in facilities with proper ventilation, systematic sanitation, ad libitum feeding, light-cycle conditions, and temperature and humidity control.

### 2.2. Production of Chimeric Antigens

**Figure 1:**
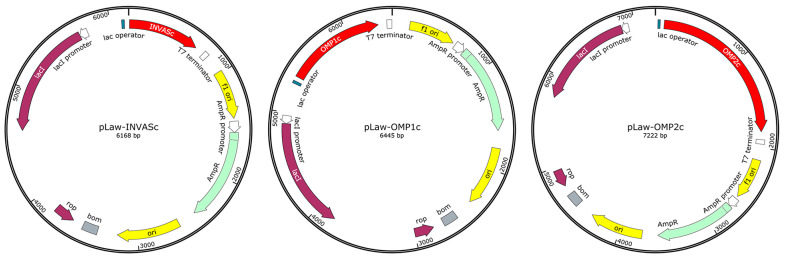
Scheme of the expression vectors pLaw-INVASc, pLaw-OMP1c, and pLaw-OMP2c. Chimeric antigens were expressed in *E. coli* under the control of the T7 promoter/T7-lacO operator and the T7 transcriptional terminator. Antigen sequences have in their C-terminal a 6XHis tag for easier identification and purification when produced in their soluble forms. Abbreviations: rop, regulatory sequence of DNA replication; bom, regulatory sequence for bacterial conjugation; LacI, lactose operon repressor; ori, plasmid replication origin; f1 ori; phage f1 replication origin.

DNA sequences of chimeric antigens were individually cloned into the expression vectors: pLawINVASc, pLawOMP1c, and pLawOMP2c (Figure 1). *E. coli* strain SHuffle^®^ T7 was transformed with the three plasmids. Individual clones of each construction were confirmed by SDS-PAGE and Western blot to further prepare master and work benches with selected clones. Ten litter batch cultures were grown in the Winpact FS-06 fermenter (Major Science, Saratoga, CA, USA), using 1 L of inoculum and 9 L of Terrific broth (TB) medium. Inoculum comprised four cultures with 250 mL Luria Bertani broth (Lioilchem, Roseto degli Abruzzi, TE, Italy) plus ampicillin 100 μg/mL (USBiological, Salem, MA, USA) (LBA). After fermenter inoculation, batch cultures supplemented with ampicillin 100 μg/mL were grown for 10 h at 37 °C under constant stirring at 150 rpm, dissolved oxygen over 20%, and pH 7.5 by the addition of 25% (*v*/*v*) NH_3_OH or 20% (*v*/*v*) H_3_PO_4_ [31]. Recombinant antigens were induced at an optical density (OD) of around 1, using 0.75 mM isopropyl β-d-1-thiogalactopyranoside (IPTG) (Santa Cruz Biotechnology, Santa Cruz, CA, USA) for 6 h. OD was measured at 600 nm (OD_600_) in the spectrophotometer UV-2505 (Labomed, Inc., Los Angeles, CA, USA), and the dry cell weight (DCW) was determined at 90 °C in a moisture analyzer (Radwag, Radon, Poland). One OD unit corresponded to 0.49 g DCW/L.

### 2.3. Extraction of Inclusion Bodies

Inclusion bodies were extracted as previously described [30]. Briefly, the biomass was resuspended in PBS (137 mM NaCl (Sigma-Aldrich, Saint Louis, MO, USA), 2.7 mM KCl (Sigma-Aldrich, Saint Louis, MO, USA), 10 mM Na_2_HPO_4_ (Sigma-Aldrich, Saint Louis, MO, USA), and 1.8 mM KH_2_PO_4_ (Merck, Darmstadt, Germany), pH 7.4) containing 0.1% Triton X-100 (Sigma-Aldrich, Saint Louis, MO, USA) and then lysed by mechanical cell disruption in the bead mill Dyno-mill ML (Wab, Muttenz/Basel, Switzerland). The pellet was separated by centrifuging at 4342× *g* for 20 min and washed twice in 1 M NaCl and 1% Triton X-100 and once in 0.05 M Na_2_HPO_4_, pH 12.0. The insoluble fraction containing inclusion bodies was resuspended in PBS supplemented with thimerosal 0.1 mg/mL and gentamicin 0.065 mg/mL to be stored at −20 °C until use. Quantification was performed using Odyssey Imaging System (LI-COR, Biosciences, Lincoln, NE, USA) after applying protein samples into SDS-PAGE and bovine serum albumin as the standard.

### 2.4. SDS-PAGE and Western Blot

SDS-PAGE analysis was performed as described by Laemmli in 1970 [32], using 12% polyacrylamide gels. For Western blot, proteins were transferred to nitrocellulose membranes (Schleicher and Schuell, Dassel, Germany), using semi-dry electroblotting equipment (TransBlot-Turbo, Bio-Rad, Hercules, CA, USA). Monoclonal mouse anti-His antibody (Cat. No. 631212, Clontech Laboratories, Mountain View, CA, USA) was used as the primary antibody, and goat Alexa Fluor^®^ 680 anti-mouse antibody (Cat. No. 115-625-146, Jackson Immuno Research, West Grove, PA, USA) was used as the secondary antibody. Infrared signals were detected using an Odyssey System (LI-COR Biosciences, Lincoln, NE, USA).

### 2.5. Microbiological Evaluation of L. intracellularis Antigens

Microbiological control was carried out by seeding the antigen mixture undiluted and 1/10 diluted in different culture media, such as Tryptone Soy Agar (TSA) (Merck, Darmstadt, Germany) for aerobic bacteria; Thioglycolate Broth (TGB) (Merck, Darmstadt, Germany) for strictly anaerobic, facultative anaerobic, and microaerophilic bacteria; and Potato Dextrose Agar (PDA) (Liofilchem, Roseto degli Abruzzi, TE, Italy) for fungi. TSA plates and TGB tubes were incubated for 7 days at 37 °C, and PDA plates were incubated at 25 °C for the same time. All cultures were performed in duplicates. The manipulation was carried out in a biosafety cabinet (AirScience, Fort Myers, FL, USA).

### 2.6. Vaccine Formulation

Chimeric antigens as inclusion bodies were mixed (1:1:1) and diluted in PBS, thimerosal 0.1 mg/mL (Sigma-Aldrich, Saint Louis, MO, USA), and gentamicin 0.065 mg/mL (Veterquímica, Santiago de Chile, Región Metropolitana, Chile) to a final concentration of 0.5 mg/mL. The antigen mixture was heated at 65 °C for 15 min and emulsified in Montanide ISA 660 VG (Seppic, Paris, France) at an antigen-adjuvant ratio of 40:60, following the manufacturer’s instructions. Emulsions of the vaccine candidate were prepared in a rotor–stator homogenizer Ultra Turrax T25 (IKA, Staufen in Breisgau, Germany) at a speed of 8000 rpm for 6 min. Additionally, an emulsion containing PBS was prepared as a negative control. Vaccine candidate formulations were packed in 50 mL glass bottles, capped with nitrile rubber stoppers, sealed with aluminum lids, and stored at 4 °C.

### 2.7. Mechanical and Thermal Stability of Formulations

All formulations were evaluated according to their thermal and mechanical stability as quality markers. To determine the mechanical stability, three tubes of 15 mL (Thermo Fisher Scientific, Waltham, MA, USA) were filled with 10 mL of samples for measuring the initial height (Ho). After centrifuging at 1512× *g* for 1 h, the final height (Hu) was measured. After one hour of centrifugation, there should be no aqueous liquid at the bottom of the tube, and an oily separation will only be allowed where the Hu/Ho height ratio is greater than 0.80 (Hu/Ho > 0.80). Thermal stability was also monitored in triplicate by incubating in a humid chamber at 37 °C in a vertical position for 15 days. Ho and Hu were measured as above. After the incubation time, the phase separation, measured by the ratio of the height of the creamy phase to the initial time and after 15 days, is expected to be greater than 0.9 (Hu/Ho ≥ 0.90).

### 2.8. Droplet Size

The droplet size of formulations was measured using a Zetasizer Nano ZS90 (Malvern Instruments, Malvern, UK) at 25 °C. All samples were diluted with Motanide ISA 660 VG at a ratio of 1:10. Droplet size should be less than 200 nm [33,34].

### 2.9. Antigen Extraction from the Emulsions

Antigens contained within the vaccine candidate and the commercial vaccine were separated from the emulsions by adding benzyl alcohol until reaching 10% (*v*/*v*). The mixture was vortexed at room temperature (RT) for 20 min and centrifuged at 16,100× *g* for 10 min. The aqueous phase containing the proteins of interest was analyzed by SDS–PAGE [35].

### 2.10. Mice Immunization

Formulation stability was evaluated in 6-week-old female C57/BL6 mice randomly distributed into two experimental groups of 10 mice. They were subcutaneously injected with water-in-oil formulations containing the following: Group 1, *E. coli* SHuffle^®^ T7 lysate; and Group 2, 12.5 μg of chimeric antigens. Mice were immunized on days 0 and 21. Blood samples were taken through the lateral saphenous vein weekly until week six, and the serum was stored at −20 °C until further use.

### 2.11. Immunization of Pigs

The immune response was evaluated in four-week-old piglets, randomly divided into four experimental groups of 13 pigs each. Immunization was carried out with water-in-oil formulations containing the following: Group 1, PBS (negative control); Group 2, 200 μg of chimeric antigens; Group 3, 100 μg of chimeric antigens; and Group 4, commercial vaccine Porcilis^®^ Ileitis (positive control). The immunization scheme included two intramuscular injections administrated with a 19 G needle on days 0 and 21. The commercial vaccine was used according to the manufacturer’s instructions. The animals were monitored daily, looking for changes in their behavior or the appearance of clinical signs. Blood samples for serum (weekly) and PBMC isolation (weeks 6 and 14) were collected to assess humoral and cellular immune responses, respectively.

### 2.12. Humoral Immune Response

Blood samples were collected in a vacutainer with clot activator (BD, vacutainer, Franklin Lakes, NJ, USA) and centrifuged at 1600× *g* for 10 min at RT. Sera were transferred to a new tube and stored at −20 °C until use. Assays for detecting humoral immune response were carried out in flat-bottom 96-well ELISA plates (Nunc MaxiSorp™, Thermo Fisher Scientific, Waltham, MA, USA) coated with a mixture of 0.5 μg/well of every recombinant antigen solubilized in 8 M urea (Merck, Darmstadt, Germany) for 16 h at 4 °C. When evaluating the contribution of each antigen to humoral immune response, wells were coated with 0.5 μg/well of individual antigens. Plates were washed once with PBS plus 0.05% Tween 20 (PBST) and blocked with 3% skim milk in PBS for 2 h at 37 °C. For the time course of the immune response and the contribution of every antigen to the immune response, sera were diluted 1/1000, while dilutions from 1/100 to 1/128,000 were used to determine the IgG antibody titer. In all cases, sera were incubated for one hour at 37 °C. After washing, a goat anti-pig IgG polyclonal antibody conjugated to HRP (Cat. No. ab6915, Abcam, Boston, MA USA) was diluted 1/15,000 and incubated for one hour at 37 °C. A solution of o-phenylenediamine dihydrochloride (OPD) (Sigma-Aldrich, Saint Louis, MO, USA) at 0.4 mg/mL (100 μL) diluted in citrate buffer was added for signal visualization. The reaction was stopped by adding 2 M H_2_SO_4_ (50 μL/well). Absorbance was measured at 492 nm in a Sinergy^®^ HTK plate reader (BioTek, Agilent Technologies, Winooski, VT, USA). The titer was calculated as the reciprocal of the highest dilution whose absorbance (Abs) value was higher than the average Abs determined at time 0 plus three times its standard deviation (Abs + 3 SD at T0).

### 2.13. Cellular Immune Response

#### 2.13.1. Isolation of Peripheral Blood Mononuclear Cells

Cellular immune response was evaluated at week 6 and week 14 after the primary immunization. Blood samples (6 mL) were collected in K_2_EDTA tubes (BD, Vacutainer, UK) and diluted in PBS (1:1 *v*/*v*). Peripheral blood mononuclear cells (PBMCs) were isolated according to the application note of Corning^®^ Lymphocyte Separation Medium (LSM) (Corning, NY, USA). PBMCs were cultured in RPMI-1640 medium (HyClone, Cytiva, Marlborough, MA, USA) with 10% fetal bovine serum and 1% penicillin–streptomycin 16 h at 37 °C and 5% CO_2_. PBMCs were seeded at 2 × 10^6^ cells/mL in 24-well plates (500 μL/well). Then, 30 μg/mL of purified *L. intracellularis* antigens (Supplementary Figure S1) or 10 μg/mL of concanavalin A diluted in 500 μL of the same culture medium was added and incubated for 24 h at 37 °C and 5% CO_2_.

#### 2.13.2. RNA Extraction and Real-Time PCR

Unstimulated and stimulated PBMCs were transferred to 1.5 mL Eppendorf tubes (Eppendorf, Hamburg, Germany) and centrifuged at 500× *g* for 10 min. The supernatant was frozen at −20 °C for subsequent measurement of secreted IFN-γ, while total RNA was isolated from cultured cells, using a NucleoSpin^®^RNA kit (Macherey-Nagel, Düren, Germany). Total RNA resuspended in nuclease-free water (HyClone, Cytiva, Marlborough, MA, USA) was quantified by Sinergy^®^ HTK Take3 Microvolume plate reader (BioTek, Agilent Technologies, Winooski, VT, USA) and diluted with nuclease-free water to a final concentration of 200 ng/μL. The cDNA was obtained by reverse transcription, using the RevertAid First Strand cDNA Synthesis kit (Thermo Fisher Scientific, Waltham, MA, USA) according to the manufacturer’s protocol. An analysis of the relative gene expression of interferon gamma (*ifn-γ*), interleukin 12 (*il-12*), and interleukin 4 (*il-4*) was performed via real-time PCR, using an AriaMx Real-Time PCR System thermocycler (Agilent Technologies, Santa Clara, CA, USA) and a KAPA SYBR Kit FAST One-Step qRT-PCR (Kapa Biosystems, Wilmington, MA, USA). Glyceraldehyde 3-phosphate dehydrogenase (GAPDH) transcripts were used as the housekeeping marker. The following primers were used for detecting gene transcripts: *ifn-γ*, forward GAGGTTCCTAAATGGTAGCTCTGGG and reverse (GATGAGTTCACTGATGGCTTTGCG); *il-12*, forward (CGTGGCTAGTTCAAGTGGTAAG) and reverse (CAGGCCCAGGAATGTTCAAA); and *il-4*, forward (GTCTGCTTACTGGCATGTACCA) and reverse (GCTCCATGCACGAGTTCTTTCT); *GAPDH*, forward (TCGGAGTGAACGGATTTGG) and reverse (TGGGTGGAATCATACTGGAAC). Cycle conditions were 3 min at 90 °C, followed by 40 repetitions of 90 °C for 10 s and 60 °C for 20 s. RNA relative quantification was calculated by the 2^−∆∆Ct^ method [36]. 

#### 2.13.3. IFN-γ Detection

IFN-γ was detected by the ELISA Flex kit (Mabtech AB, Nacka Strand, Sweden) according to the manufacturer’s indications. Flat-bottom 96-well ELISA plates were coated with 2 μg/mL of the monoclonal antibody to porcine IFN-γ (Cat. No. 3130-3-250, Mabtech AB, Nacka Strand, Sweden) for 16 h at 4 °C. Plates were blocked with 0.1% BSA in PBST for one hour at RT. The supernatants from stimulated and unstimulated PBMCs were added for 2 h at RT. The monoclonal anti-porcine IFN-γ antibody mAb (P2C11) conjugated to biotin (1 μg/mL) (Cat. No. 3130-6-250, Mabtech AB, Nacka Strand, Sweden) was added for one hour at RT. After adding Streptavidin-HRP diluted to 1/1000 (Cat. No. 3310-9-1000, Mabtech AB, Nacka Strand, Sweden), signals were visualized with a TMB solution (Mabtech AB, Nacka Strand, Sweden). Absorbance was measured at 450 nm, using a Sinergy^®^ HTK plate reader (BioTek, Agilent Technologies, Winooski, VT, USA).

#### 2.13.4. Flow Cytometry

PBMCs were isolated, cultured, and treated as previously described in Section 2.13.1. The incubation period lasted 5 days at 37 °C and 5% CO_2_. Four experimental groups were evaluated: Group 1, untreated negative control; Group 2, treated negative control; Group 3, untreated 200 μg dose; and Group 4, treated 200 μg dose. Every group was divided into three pools to identify different cellular surface markers. For this purpose, each pool of cells was resuspended in PBS (1 × 10^6^ cells in 200 μL). The first pool was treated with 4 μL of Alexa Fluor 647 Mouse Anti-Pig CD8a (BD Pharmingen, Franklin Lakes, NJ, USA), the second pool with 4 µL of FITC Mouse Anti-Pig CD3 ε (BD Pharmingen, Franklin Lakes, NJ, USA), and the third pool with 4 μL of PE-Cy TM 7 Mouse Anti-Pig CD4a (BD Pharmingen, Franklin Lakes, NJ, USA). After 30 min of incubation at 4 °C in the darkness, cells were washed and resuspended in 250 μL PBS for further analysis in the BD FACSAria III cell sorter Flow Cytometer (BD Life Science, Franklin Lakes, NJ, USA).

### 2.14. Histopathological Analysis

Animals were sacrificed at the end of the experiments to obtain samples for histopathological assays according to the protocols described by Luna, 1968 [37]. Briefly, slides with muscle tissue of the inoculation zone were fixed in 10% formalin for 24–48 h. The samples were sectioned 5 μm thick in a Shandon Citadel 1000 tissue processor (Thermo Fisher Scientific, Waltham, MA, USA) with a vacuum pump and an inclusion center Microm A280. The cross-sections were stained with hematoxylin–eosin (Göteborg, Sweden) and observed with a microscope Axiokop 40 (Carl Zeiss, Oberkochen, Germany). Signs observed in the inoculation site were scored from 0 to 3 according to histopathologic changes: score 0, no histological alteration; score 1, few inflammatory cells (mild inflammation); score 2, 40% of the area covered with inflammatory cells (moderate inflammation), and score 3, the majority of the area covered with inflammatory cells and granuloma formation (high inflammation).

### 2.15. Statistical Analysis

Statistical analyses were performed using the software GraphPad Prism version 10.0.0 for Windows (GraphPad Software, Boston, MA, USA). The data were evaluated using parametric and non-parametric methods depending on the results of the Bartlett test for homogeneity of variances and the Shapiro–Wilk test for normality. The data transformation was also performed to improve the assumptions of the statistical tests. The statistical analysis specifications are included in figure legends. Significance was considered for *p* < 0.05.

## 3. Results

### 3.1. Expression of Chimeric Antigens and Characterization of the Formulation

**Figure 2:**
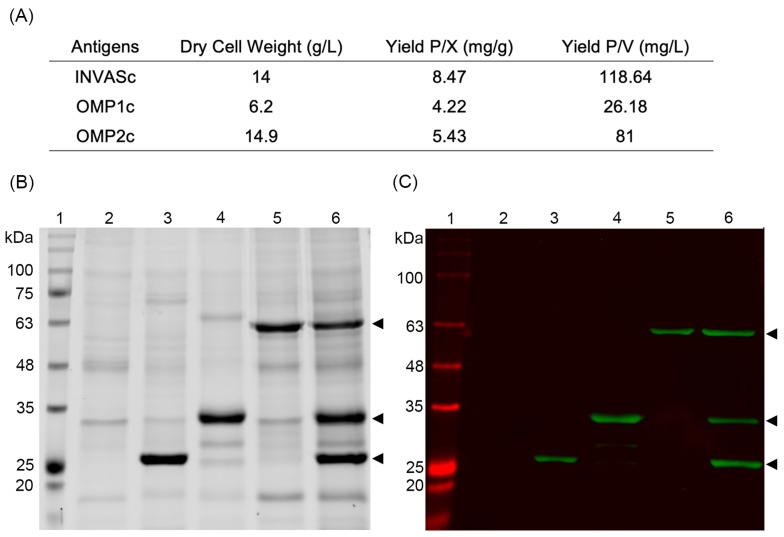
Antigen production and characterization. Chimeric antigens were expressed individually as inclusion bodies in 10 L fermentations, using the *E. coli* strain SHuffle^®^ T7 as the host. (**A**) Protein yields of INVASc, OMP1c, and OMP2c. Characterization of chimeric antigens by SDS-PAGE (**B**) and Western blot (**C**). Lane 1, molecular weight marker AccuRuler RGB Plus prestained protein ladder (Maestrogen, Taiwan); lane 2, negative control (untransformed bacteria); lane 3, INVASc; lane 4, OMP1c; lane 5, OMP2c; and lane 6, mixture of the three chimeric antigens. Arrowheads indicate the three chimeric antigens.

Formulation inconsistencies in the previous multi-antigenic vaccine candidate against *L. intracellularis* prompted us to improve how the three chimeric proteins are expressed to achieve equal antigen quantities in the final formulation with the aim of inducing a reproducible immune response for the vaccine candidate and to achieve lot-to-lot consistency during formulation. For this purpose, the recombinant genes OMP1c, OMP2c, and INVASc were cloned and expressed individually in 10 L fermentations. Although the production of OMP2c protein showed the highest cell biomass (14.9 g/L), there was more antigen per dry cell biomass during INVASc production (8.47 mg/g), resulting in the best antigen yield (118.64 mg/L). In the case of OMP1c protein, the expression system could only reach a total yield of 26.18 mg/L, showing the lowest production levels (Figure 2A). After extracting inclusion bodies, the SDS-PAGE and Western blot showed protein integrity and the expected band pattern for INVASc (25 kDa), OMP1c (35 kDa), and OMP2c (65 kDa). Antigen quantification delivered around 60% in the mixture (Figure 2B,C), and microbiological studies of the antigen mixture did not show fungal or bacterial contamination.

**Figure 3:**
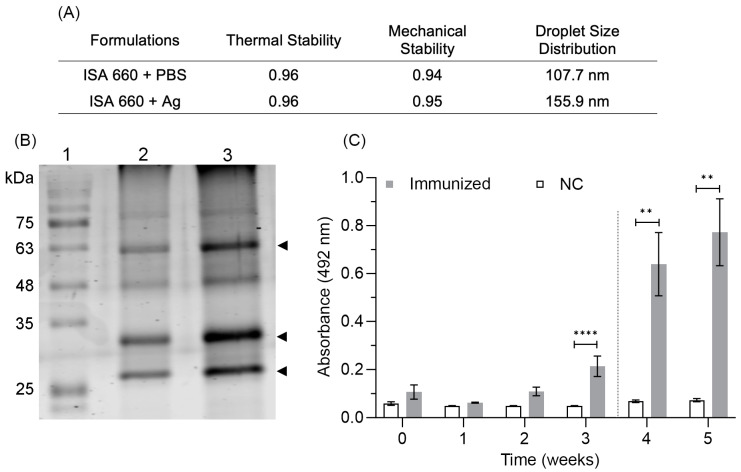
Stability characterization of chimeric antigens formulated in Montanide ISA 660 VG. (**A**) Physical characterization of emulsions containing PBS and the three antigens. (**B**) SDS-PAGE of chimeric antigens after being extracted from emulsions formulated three months, previously. Arrowheads indicate the three chimeric antigens. (**C**) Antigen stability assay in mice using formulations incubated at 4 °C for three months. NC: negative control (PBS+Montanide). The dashed line indicates the booster. The mean and the standard error correspond to 10 mice. The statistical significance at different times was determined using the Friedman test, followed by Dunn’s multiple comparison test. The statistical significance between groups at each time was determined using the Mann–Whitney test; ** *p* ≤ 0.01, and **** *p* ≤ 0.0001.

The formulation comprising the adjuvant Montanide ISA 660 VG emulsified with the aqueous phase containing the three chimeric antigens showed mechanical and thermal stability, along with height ratios (Hu/Ho) superior to 0.8. The average droplet diameter between 100 and 160 nm also demonstrated sample cohesion (Figure 3A). After three months of storage at 4 °C, the antigen integrity remained intact within the formulation (Figure 3B), as well as their properties to stimulate the immune system. Mice immunized with the stored formulation induced a humoral immune response with absorbance values above 0.6 after the booster, significantly superior to negative controls and absorbance values obtained after the first immunization (Figure 3C).

### 3.2. Immune Response in Pigs

The previous antigen production and characterization revealed high protein yields and proper antigen stability and functionality within the formulation in vitro and in vivo. Then, we evaluated the antigen immunogenicity in pigs to confirm the induction of an appropriate humoral and cellular response in the target species and corroborate the former results in mice.

#### 3.2.1. Humoral Immune Response

**Figure 4:**
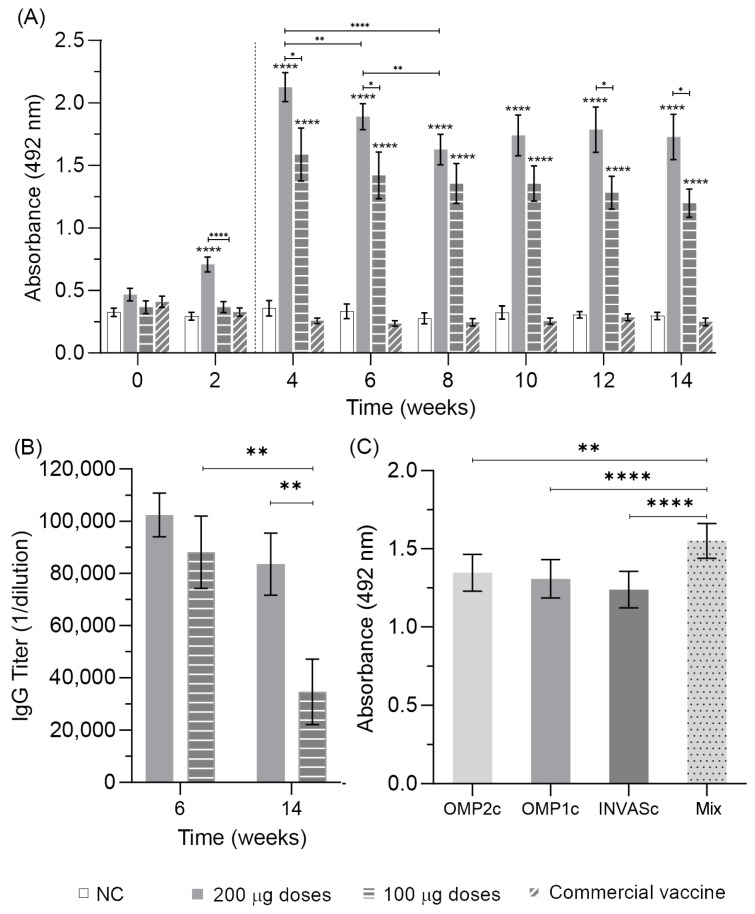
Humoral immune response induced by the vaccine candidate in pigs. (**A**) Indirect ELISA measuring antibody values in the pig sera diluted 1/1000 after being immunized with formulations containing PBS (NC: negative control), the three chimeric antigens (vaccine candidate) at different doses, and the commercial vaccine Porcilis^®^ Ileitis as a positive control. The dashed line indicates the booster. The mean and the standard error correspond to 13 pigs. The statistical significance among different times was determined by the one-way ANOVA, followed by the Bonferroni test for multiple comparisons; ** *p* ≤ 0.01, and **** *p* ≤ 0.0001. The statistical significance between groups at each time was determined by the one-way ANOVA followed by the Bonferroni test for multiple comparisons; * *p* ≤ 0.05, and **** *p* ≤ 0.0001. Significances without lanes correspond to differences among the negative control and the commercial vaccine groups. (**B**) IgG antibody titer induced by vaccine formulations at different doses and times. The statistical significance among different times was determined by the Wilcoxon matched-pairs signed-rank test; ** *p* ≤ 0.01. The statistical significance between groups of each time was determined by the Mann–Whitney test; ** *p* ≤ 0.01. (**C**) The contribution of each antigen to the IgG humoral immune response was determined by indirect ELISA. The statistical significance was assessed by the ANOVA test followed by the Bonferroni test for multiple comparisons; ** *p* ≤ 0.01, and **** *p* ≤ 0.0001.

The first immunization in pigs induced a significant antibody production at week two with 200 μg of the vaccine candidate (Figure 4A). After the booster, antibody levels abruptly increased in the two groups immunized with the vaccine candidate, showing significant differences with positive and negative control groups. Antibody levels of pigs immunized with 200 μg were significantly higher than their counterparts of 100 μg at weeks 4, 6, 12, and 14. Only in weeks 8 and 10, there were no significant differences in antibody levels between the two doses administered. Remarkably, the ELISA assay coating with the chimeric antigens did not recognize antibodies in the sera of pigs immunized with the commercial vaccine Porcilis^®^ Ileitis as a positive control, and the antibody detection in this experimental group was on par with the negative control during the entire experiment.

Antibody titration was performed at weeks 6 and 14, using the sera of pigs immunized with 100 and 200 μg of the antigen formulation (Figure 4B). Sera from week 6 showed antibody titers of 1/102,400 (log_2_ = 17) and 1/83,464 (log_2_ = 15) for doses of 200 and 100 μg, respectively. Meanwhile, at week 14, the same doses showed titers of 1/88,067 (log_2_ = 16) and 1/34,614 (log_2_ = 13). There were no significant differences in titer values between doses at the same time. However, a significant difference was observed when comparing the 100 μg dose between the two weeks.

The individual contribution of chimeric antigens to the humoral immune response was measured to know if there was any kind of antigenic immunodominance during the activation of the immune system by the vaccine candidate. High antibody levels were observed for the three chimeric antigens, without any significant difference among the results (Figure 4C), demonstrating that humoral response induced upon vaccine candidate administration produces similar specific antibody levels for the three antigens.

#### 3.2.2. Cellular Immune Response

Intracellular pathogens like *L. intracellularis* often induce an effector response mediated by T lymphocytes to eliminate infected cells and eradicate the disease. Here, we used indirect and direct methodologies to confirm the induction of a cellular immune response against the three chimeric antigens.

##### Cytokine Detection by qPCR and Indirect ELISA

**Figure 5:**
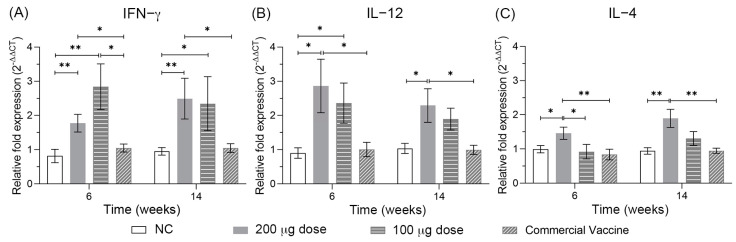
Cytokine analysis via real-time PCR. Transcripts were detected from peripheral mononuclear blood cells (PBMCs) of animals from every experimental group at weeks 6 and 14. PBMC stimulation was performed with solubilized, purified, and renatured *L. intracellularis* antigens. Unstimulated PBMCs were used as controls. Data are expressed as the means and the standard error from 8 determinations. Relative expression of IFN-γ (**A**), IL-12 (**B**), and IL-4 (**C**) transcripts. The statistical significance among different times was determined by the Wilcoxon matched-pairs signed-rank test. The statistical significance between groups at each time was performed by the Kruskal–Wallis test, followed by the Mann–Whitney test adjusted for multiple comparisons with the Benjamini–Hochberg method; * *p* ≤ 0.05, and ** *p* ≤ 0.01.

Transcripts of cytokines IFN-γ, IL-12, and IL-4 were determined by qPCR at 6 and 14 weeks after the first immunization, as markers of cellular immune response. Lymphocytes isolated from animals immunized with 200 and 100 μg of the vaccine candidate and stimulated with solubilized and purified *L. intracellularis* antigens showed a significant increase in IFN-γ expression. At week 6, IFN-γ values were higher for the dose of 100 μg than those obtained for 200 μg. By week 14, IFN-γ values were almost similar for both doses (Figure 5A); however, there were no significant differences between doses at both times that were evaluated.

Although IL-12 transcripts showed a higher level at 200 μg dose than the values obtained for 100 μg dose at both times measured, there were no significant differences between them (Figure 5B). Meanwhile, the IL-4 transcript levels were lower than those obtained for IFN-γ and IL-12 (Figure 5C). We did not detect cytokine expression in lymphocytes isolated from animals immunized with the commercial vaccine.

**Figure 6:**
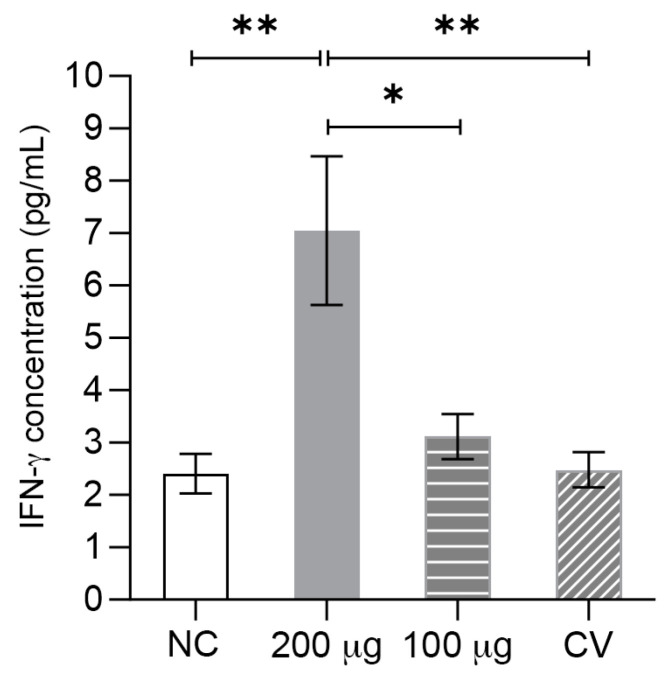
Determination of the IFN-γ cytokine by the ELISA Flex kit. PMBCs from every experimental group at week 14 were stimulated with solubilized, purified, and renatured *L. intracellularis* antigens. Unstimulated PBMCs were used as controls. After stimulation, supernatants were collected for IFN-γ detection. NC: negative control. CV: commercial vaccine. Data are expressed as the mean and standard error of 13 pigs. Statistical significance was determined by the Kruskal–Wallis test, followed by the Mann–Whitney test adjusted for multiple comparisons with the Benjamini–Hochberg method; * *p* ≤ 0.05, and ** *p* ≤ 0.01.

IFN-γ is a crucial cytokine in the Th1 cellular response, which induces the removal of infected cells with the intracellular pathogen. As IFN-γ transcript levels were significantly increased in the longer time evaluated (14 weeks) during the previous assay, we also measured IFN-γ cytokine levels in the supernatant of lymphocyte cultures with a commercial ELISA kit. The results revealed a significant increase in IFN-γ levels at a 200 μg dose, with values around 7 pg/mL (Figure 6). No levels of IFN-γ were detected in the commercial vaccine group.

##### Lymphocyte Proliferation

**Figure 7:**
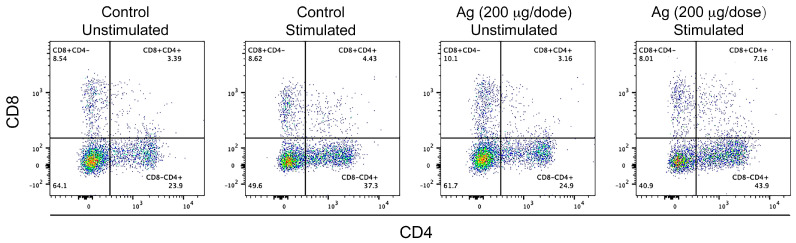
Lymphocyte proliferation analysis by flow cytometry. PMBCs from the experimental group immunized with 200 μg of chimeric antigens at week 14 were stimulated with solubilized, purified, and renatured *L. intracellularis* antigens. Unstimulated PBMCs were used as controls. Isolated cells were treated with Alexa Fluor 647 Mouse Anti-Pig CD8a, FITC Mouse Anti-Pig CD3 ε, and PE-Cy TM 7 Mouse Anti-Pig CD4a for identifying distinct lymphocyte populations. Results are based on the count of more than 10,000 events. Values represent the measurements of 3 pools of 4 animals for the two experimental groups.

Abrupt lymphocyte proliferation and differentiation upon antigen encounter characterize a cellular immune response. Therefore, we evaluated the proliferation of TCD4 and TCD8 lymphocyte populations, using the samples of animals immunized at week 14 with a dose of 200 μg after being stimulated with the same mixture of *L. intracellularis* antigens used in the preceding assays. After counting 10,000 events for each experimental group via flow cytometry, a substantial increase in TCD4^+^CD8^−^ and TCD4^+^CD8^+^ lymphocyte populations was observed in the experimental group with the 200 μg dose stimulated with *L. intracellularis* antigens compared with the unstimulated group and negative controls (Figure 7). This result suggests that mainly TCD4^+^ lymphocytes could be responsible for the pattern of cytokine expression observed before, constituting an important stimulus for the TCD8^+^ population to activate and eliminate infected cells.

### 3.3. Safety Evaluation of the Vaccine Candidate

**Figure 8:**
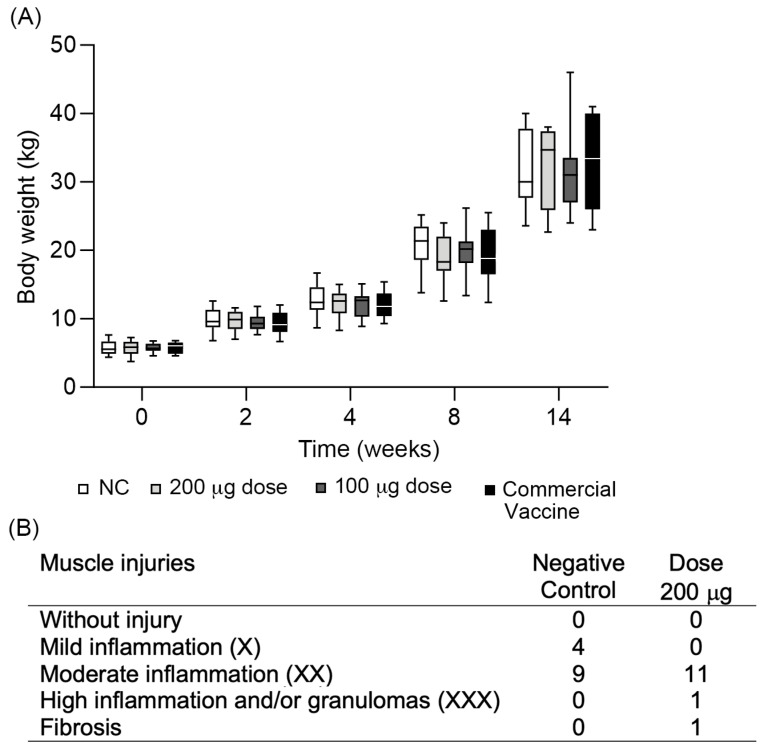
Evaluation of the vaccine candidate on animal health after immunization. (**A**) Average daily weight of pigs after the immunization with the new vaccine candidate against *L. intracellularis*. NC: negative control. Values represent the mean and the standard error of 13 pigs. Statistical significance was determined by one-way ANOVA between groups at each time. (**B**) Injury evaluation of the inoculation site after the histopathological analysis was performed by scoring tissue samples according to the presence of inflammatory cells in the analyzed area.

During the immunization experiment in pigs, body weight, changes in animal behavior, fever, weight loss, diarrhea, and other clinical manifestations that could indicate an adverse reaction to the vaccine candidate were evaluated. A steady increase in animal weight was observed with no significant difference between experimental groups (Figure 8A). There were no behavioral changes, diarrhea, or variation in the consistency and color of feces, thus indicating that no *L. intracellularis* infection occurred during the experiment.

Macroscopic alterations in the inoculation site of animals were not observed, but the histopathological analysis of the muscular tissue of most animals showed moderate inflammatory reactions. These lesions were mainly attributed to the adjuvant Montanide ISA 660 VG because they were also observed in animals of the negative control group (Figure 8B). No histopathological alterations occurred in the inoculation site of pigs immunized with the commercial vaccine. High inflammatory reactions were observed only in two pigs immunized with the vaccine candidate. No signs of infection and structural changes were observed.

## 4. Discussion

Most farms with intensive pig production worldwide usually have a high prevalence of *L. intracellularis* [38,39,40]. Periodic outbreaks of this intracellular pathogen severely affect animal health and profits in this relevant economic area [41]. Vaccination has demonstrated high efficacy in eradicating and offering immune protection against infectious diseases for centuries. Current methods for activating the immune system against *L. intracellularis* involve re-infection with the pathogenic bacteria [42,43] or vaccination with the two approved commercially available vaccines: the live-attenuated vaccine, Enterisol^®^; and the inactivated vaccine Porcilis^®^, Ileitis [12,14,44]. Considering that these vaccines do not offer a total protection level and the intrinsic disadvantages they have, in which the high demand on the in vitro culture and proliferation of the pathogen to obtain the active principle within them is a major hurdle, further studies using new vaccine approaches need to be performed to develop more effective vaccine candidates to counteract this disease. Our investigation group has successfully designed and produced a subunit vaccine candidate against *L. intracellularis* based on three engineered proteins from the pathogen [29].

Nevertheless, uneven antigen amounts in the vaccine candidate formulation could be problematic from biological and practical points of view. Initially, our production system synthesized the three chimeric antigens simultaneously with different expression levels. Under these conditions, it was challenging to obtain formulations with equal antigen amounts. This unfavorable outcome could induce an imbalanced immune response against antigens, affecting the protection degree of immunized animals. In this study, we produced the chimeric antigens separately by cloning the three genes individually in the same expression vector (Figure 1), intending to obtain more homogeneous vaccine formulations by mixing equal antigenic quantities.

The production of individual antigens in *E. coli* under standard conditions allowed for the development of in vitro and in vivo experiments. As in a previous study, we obtained a 70-fold increase in protein yields when expressing the three antigens simultaneously by adjusting medium and culture conditions [30]. It is highly probable that a similar optimization applied to the individual antigen production also provides a significant improvement of antigen yields.

Subunit vaccines usually include an adjuvant to potentiate the specific immune response against vaccine antigens. We prepared our vaccine candidate by using the adjuvant Montanide^TM^ ISA 660 VG, which generates water-in-oil formulations with continuous oil-phase emulsions. These kinds of adjuvants induce efficient and long-term humoral and cellular immune responses. They are also compatible with inactivated and recombinant antigens (https://www.seppic.com/en/montanide-isa-w-o, accessed on 27 July 2023). Stabilizing the oily and aqueous phases during the emulsion favors the generation of tiny droplets. The size of these droplets can deeply influence the fate of the immune response. The droplet size we obtained in this study upon adjuvant emulsion agrees with some research, where droplet sizes under 200 nm and over 70 nm of vaccine formulations increased the immune response [33,34].

A favorable immune response pattern was observed with our new vaccine candidate formulated with even antigen amounts. These results were predictable due to outcomes with a similar vaccine candidate [29,30]. However, we obtained higher antibody titers and a superior cellular response in this study. The significant increase in IFN-γ and IL-12 suggests a cellular response with a Th1 pattern, which is the type of lymphocyte response needed to eliminate intracellular pathogens, just like *L. intracellularis*. 

Surprisingly, no immune response was observed when pigs were immunized with the commercial vaccine Porcilis^®^ Ileitis. This unexpected result could be due to several issues: (i) low antigen representation in the commercial vaccine and (ii) low immunogenicity of native antigens could severely compromise the immune response in a way that indirect ELISAs may not detect it; (iii) specific antigenic divergences between the *L. intracellularis* strain of commercial vaccine (SPAH-08) and the strains from which we selected the three antigens for our vaccine candidate (PHE/MN1-00 and N343), despite the high homology described among *L. intracellularis* isolates by analyzing 16S ribosomal DNA gene and targeting outer membrane proteins with polyclonal and monoclonal antibodies [45,46]; and (iv) ELISAs for detecting the humoral response were coated with denatured antigens in 8M urea, meaning that antigenic recognition would be limited to lineal epitopes. If the commercial vaccine used in the immunization assay mainly induces antibodies that recognize conformational epitopes, the humoral response detection would be almost null using denatured antigens in ELISA procedures. Taking into account this last consideration, the humoral response detected in this study would be mainly by binding antibodies to linear epitopes. To know the involvement of conformational epitopes in the induction of the humoral response, the three chimeric antigens were solubilized, purified, and renatured (Supplementary Figure S1). These novel antigens were recognized by the sera of experimental groups immunized with the vaccine candidate and the commercial vaccine at the last time evaluated (week 14) in Western blot assays (Supplementary Figure S2). ELISA assay with renatured antigens also showed a significantly higher antibody response with the sera of the experimental group immunized with the vaccine candidate; still, the sera of the commercial vaccine group do not show significant differences compared to the negative control (Supplementary Figure S3). Therefore, antibodies induced by our vaccine candidate are specific for the three chimeric antigens in the formulation and recognize linear and conformational epitopes. The low antibody recognition in the sera of the commercial vaccine group suggests that the unresponsiveness of this experimental group could be related to the first three issues mentioned above because this vaccine (Porcilis^®^ Ileitis) has also demonstrated the induction of a protective immune response [12]. Likewise, our recombinant subunit vaccine candidate has shown signs of protection by histopathological analyses [29].

After immunization and in the time course of animal experimentation, the vaccine candidate proved to be safe for pigs. Although some moderate muscular lesions occurred at the injection site, no symptoms indicated adverse effects on animal health.

## 5. Conclusions

This study demonstrated the promising immunological properties and safety of a new vaccine candidate against the intracellular pathogen *L. intracellularis*. Considering the important economic losses that proliferative enteropathy causes in the swine industry and the disadvantages of currently approved vaccines to counteract this disease, the development of new-generation efficient vaccines with easier, faster, and cost-effective production systems is highly relevant. Our vaccine candidate showed promising results and has the added value of obtaining the desired antigens as inclusion bodies, thus allowing us to avoid additional steps for protein purification with chromatographic methods and subsequent renaturation.

## Data Availability

The data presented in this study are available on request from the correspondent author.

## Supplementary Materials

The following supporting information can be downloaded at https://www.mdpi.com/article/10.3390/vaccines11121817/s1, Figure S1: Purification process of soluble chimeric antigens from *L. intracellularis*. Figure S2: Western blot assays confronting soluble chimeric antigens to the sera of the negative control group (A), the vaccine candidate group (B), the commercial vaccine group (C). Figure S3: Antibody detection using soluble chimeric antigens from *L. intracellularis*. Indirect ELISA was done by coating the 96 well plates with 0.5 μg/well of the antigen mixture and sera from the negative control group (NC), the commercial vaccine group (CV), and the vaccine candidate group (VC) were evaluated at a dilution 1/200.
