# Supplementary material for: New Formulation of a Subunit Vaccine Candidate against Lawsonia intracellularis Increases Humoral and Cellular Immune Responses
Source: Vaccines (Basel). 2023 Dec 4;11(12):1817. doi: 10.3390/vaccines11121817 (PMC10747550; doi:10.3390/vaccines11121817)
Supplement: Supplementary file 1 [file vaccines-11-01817-s001.zip › vaccines-2598579-supplementary.pdf]

Suplementary material

The objectives of obtaining the chimeric antigens from *L. intracellularis* in their soluble form were to stimulate PBMC for evaluating the cellular immune response and to determine if antibodies on sera from immunized pigs also recognized conformational epitopes.

Material and methods

Antigen solubilization

Inclusion bodies from the three chimeric antigens were centrifuged at 12000 x g for 15 min, and the pellet was resuspended in a solubilization buffer (50 mM sodium phosphate, 300 mM NaCl, 5 mM imidazole, 0.1 % SDS, pH 7.4) after discarding supernatants. Solutions were homogenized for 10 min, centrifuged at 12000 x g for 15 min, and frozen at 4 °C for one hour. The SDS excess was precipitated by centrifuging twice at 12000 x g for 30 min at 4 °C. Clear supernatants were collected in new tubes.

Antigen purification

Solubilized antigens were purified by immobilized metal-affinity chromatography (IMAC). Solutions containing solubilized antigens were supplemented with 5 mM imidazole (Merck, Germany), pH 8.0, and passed through 0.45 µm pore size filters before being added to a column filled with 5 mL of the IMAC Sepharose 6 Fast Flow matrix (GE Healthcare, Sweden), which had previously been loaded with a solution of 0.25 M $NiSO_4$ (Merck, Germany) and equilibrated with a buffer containing 50 mM sodium phosphate, 300 mM NaCl, 5 mM imidazole, 0.01% SDS, pH 7.4 at a flow rate of 1 mL/min. The wash was performed with five volumes of the previous buffer containing 25 mM imidazole, and chimeric antigens were eluted with the same buffer containing 100 mM imidazole. Fraction detection was performed using ÄKTA prime view software and an ÄKTA prime plus chromatography station (GE Healthcare, Sweden). After purification, chimeric antigens were dialyzed in a collecting buffer (50 mM sodium phosphate, 300 mM NaCl, 0.01% SDS, pH 7.4) and concentrated in a 10 kDa centricon. Protein concentration was determined by a BCA protein assay kit (Pierce, USA) using BSA as a calibration standard. Aliquots of 1 mL were stored at -20 °C until use.

SDS-PAGE and Western blot

These techniques were done as described in Materials and Methods (section 2.4) of the main text.

Indirect ELISA for detecting antibodies specific for the chimeric antigens

The general procedure of this indirect ELISA was done as described in Materials and Methods (section 2.12) of the main text, with some modifications. (i) ELISA plates were coated with a mixture of solubilized, purified, and renatured chimeric antigens at the same concentration previously used, and (ii) sera from the three experimental groups and naturally infected pigs were used at a dilution 1/200.

Results

Antigen purification was done by affinity chromatography, which often gives a high purity degree of the desired proteins in only one chromatographic step. After antigen solubilization, purification was performed by Immobilized Metal Affinity Chromatography (IMAC), taking advantage of histidine tags included in the three chimeric antigens during the design of their nucleotide sequences.

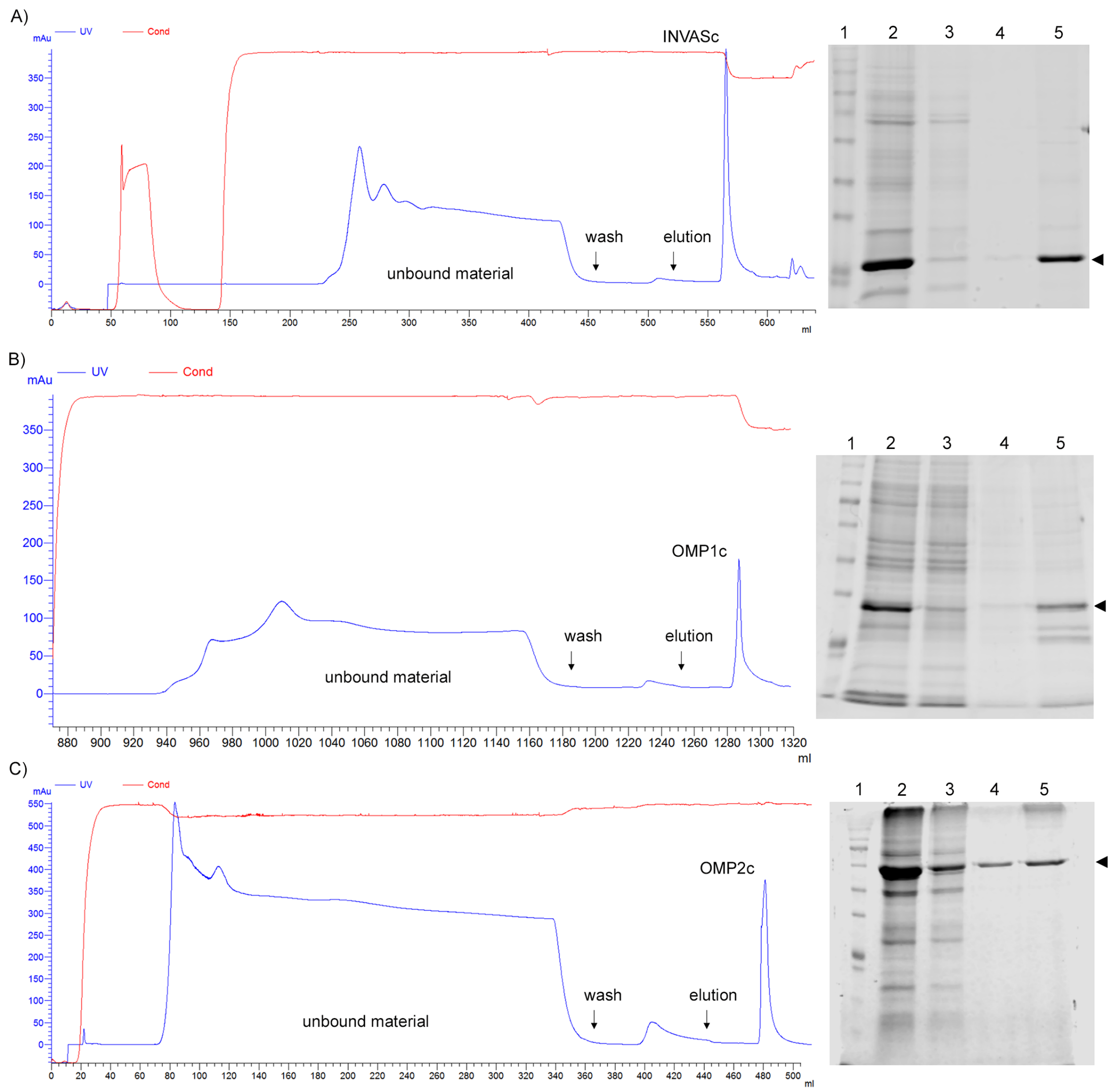

Figure S1: Purification process of soluble chimeric antigens from *L. intracellularis*. Chromatograms and SDS-PAGE correspond to the IMAC procedure for purifying the chimeric antigens INVASc (A), OMP1c (B), and OMP2c (C). 1: AccuRuler RGB Plus prestained protein ladder (Maestrogen, Taiwan), 2: Initial sample, 3: Unbound material, 4: Wash, 5: Elution. Arrowheads indicate the proteins of interest.

Most impurities were eliminated in the unbound material, and a little protein loss was observed in the wash. The elution step recovered the majority of desired proteins with a purity degree between 80 and 90% (Fig. S1). After purification and renaturation, the amount and purity of chimeric antigens allowed us to perform protein immune identification and antigenic recognition experiments.

In previous experiments, we only observed antigen recognition by antibodies in the sera of pigs immunized with our vaccine candidate, but not by those of pigs immunized with the commercial vaccine Porcilis® Ileitis when used denatured antigens with urea 8 M in ELISA assays. One of our hypotheses was based on the lack of antibody recognition in the experimental group immunized with the commercial vaccine by the scarcity of conformational epitopes, assuming that most of the humoral response induced by this commercial vaccine generates antibodies against this kind of epitopes rather than the linear ones. In western blot assays using fluorescent antibodies, signals were observed in the sera of pigs immunized with our vaccine candidate and the commercial vaccine.

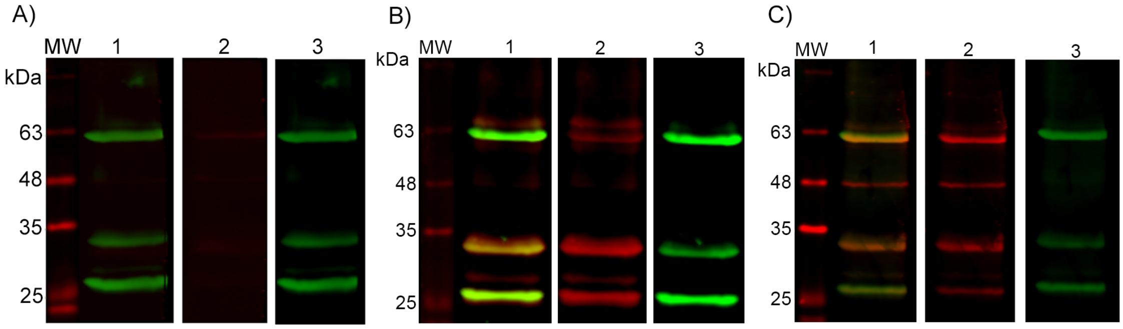

Figure S2: Western blot assays confronting soluble chimeric antigens to the sera of the negative control group (A), the vaccine candidate group (B), the commercial vaccine group (C). 1: Molecular weight marker. 2: Double channel detection at 680 nm and 790 nm, 3: Pig's sera diluted 1:100 as primary antibodies and a goat anti-swine IgG conjugated to Alexa fluor 647 as the secondary antibody, 4: Monoclonal mouse anti-Histidine Tag as the primary antibody and a goat anti-mouse IgG conjugated to Alexa fluor 790 nm as the secondary antibody.

This result demonstrated the presence of antigen-specific antibodies in all sera evaluated. However, the indirect ELISA showed no significant differences between absorbance values (Abs) of the commercial vaccine group and the negative control, while significantly high antibody level in the group of our vaccine candidate were observed.

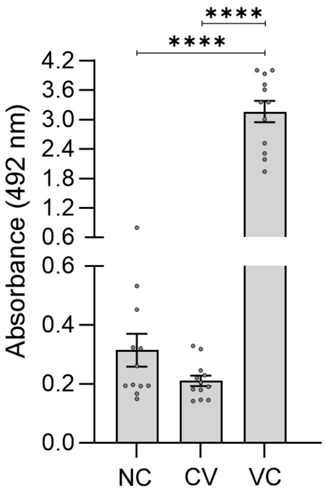

Figure S3: Antibody detection using soluble chimeric antigens from *L intracellularis*. Indirect ELISA was done by coating the 96 well plates with 0.5 μg/well of the antigen mixture and sera from the negative control group (NC), the commercial vaccine group (CV), and the vaccine candidate group (VC) were evaluated at a dilution 1/200. The mean and standard error corresponded to 13 pigs. Statistical significance was determined by the Kruskal-Wallis test, followed by the Dunn's multiple comparisons test. **** $p \leq 0.0001$.

Overall, we concluded that our vaccine candidate induces a humoral response able to generate antibodies with a high recognition level of linear and conformational epitopes, whereas chimeric antigens were poorly recognized by the sera of pigs immunized with the commercial vaccine.

The results corresponding to densitometry readings/intensity ratio of each band in the Western blot figures are shown. Also, we included the whole blot (uncropped blots) showing all the bands with all molecular weight markers on the Western.

Image ID: 0026017_09
Acquire Time: May 30, 2023 2:43:28 PM

Acquisition Information

#	Image ID	Acquire Time	Channels	Resolution	Intensities	Quality	Analysis	Image Name
1	0026017_09	May 30, 2023 2:43:28 PM	700 800	84um	Auto Auto	high	Manual	Figure 2 B

Image Display Values

Channel	Color	Minimum	Maximum	K
700	Gray Scale (Black on White)	0.282	4.57	0

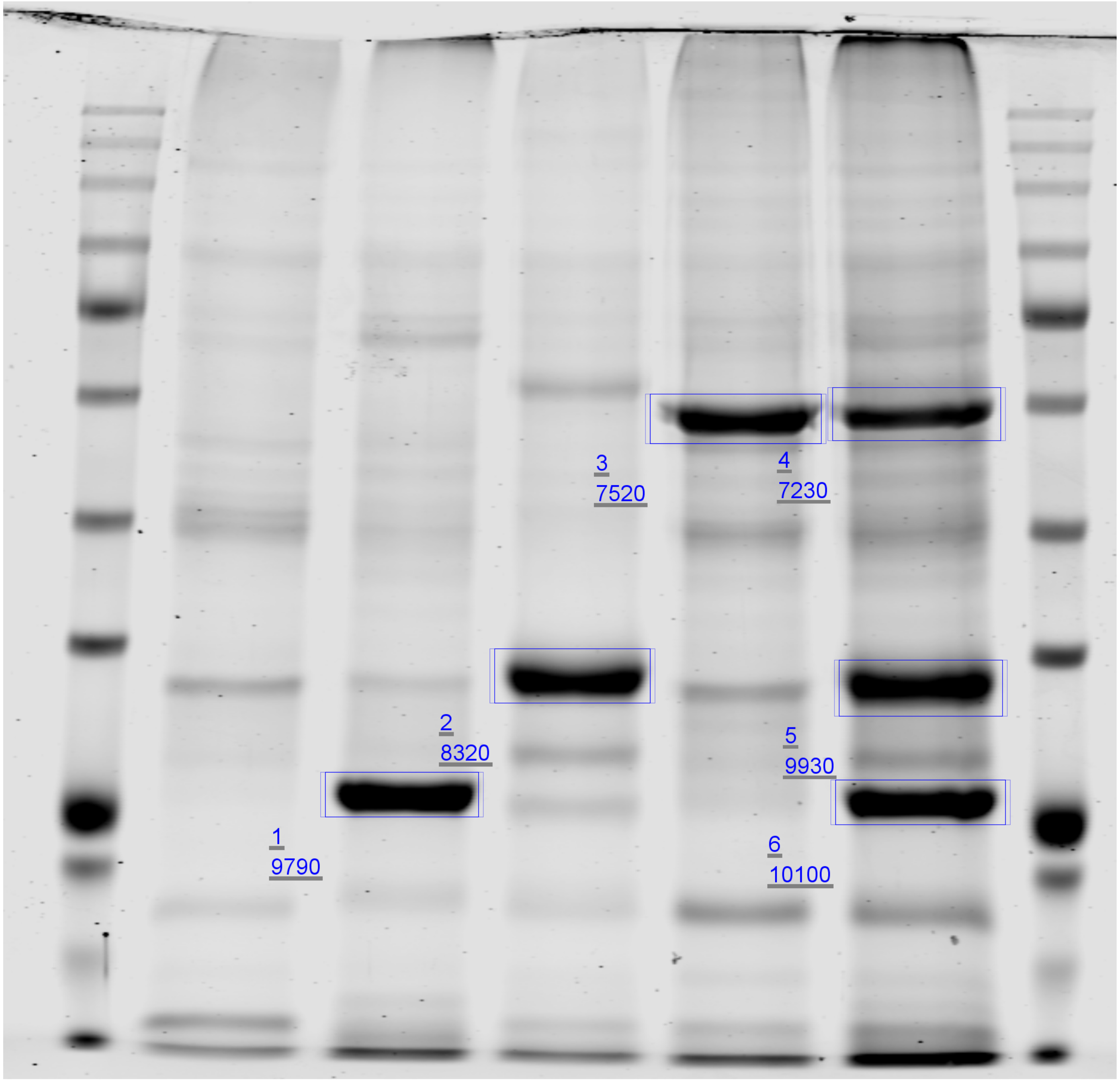

Image ID: 0026017_09
Acquire Time: May 30, 2023 2:43:28 PM

Acquisition Information (continued)

#	Comment	Image Modifications
1	Lawsonia antigens SDS PAGE	Flip Top to Bottom Image ID: 0026017_01; Flip Right to Left Image ID: 0026017_02

Image ID: 0026017_09
Acquire Time: May 30, 2023 2:43:28 PM

Acquisition Information

#	Image ID	Acquire Time	Channels	Resolution	Intensities	Quality	Analysis	Image Name
1	0025941_01	May 25, 2023 4:55:51 PM	700 800	169um	Auto Auto	medium	Manual	Figure 2 C

Image Display Values

Channel	Color	Minimum	Maximum	K
700	Red	0.335	77.7	0
800	Green	5.71	15.0	0

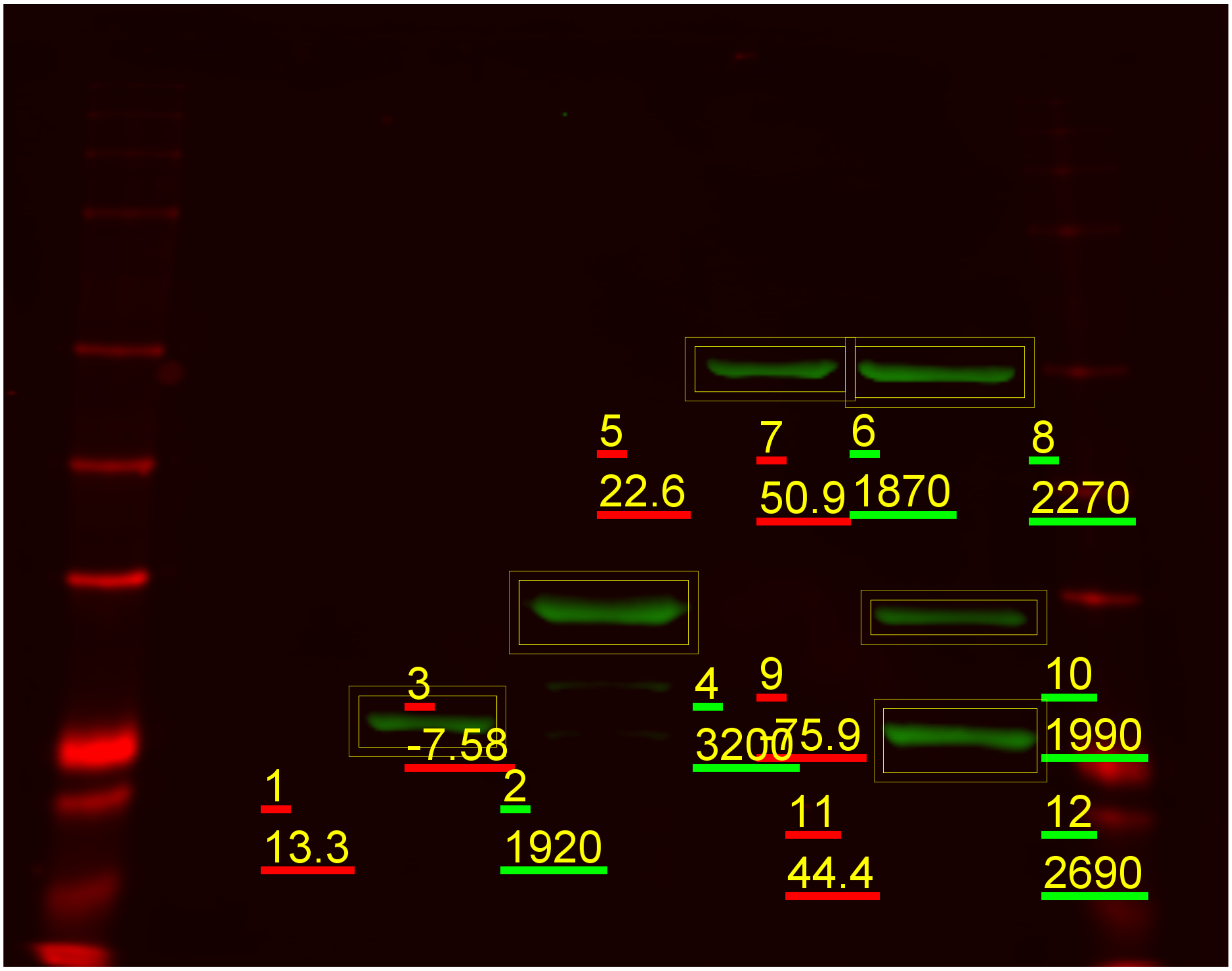

Image ID: 0026017_09
Acquire Time: May 30, 2023 2:43:28 PM

Acquisition Information (continued)

#	Comment	Image Modifications
1	Lawsonia antigens Western blot	

Image ID: 0026017_09
Acquire Time: May 30, 2023 2:43:28 PM

Acquisition Information

#	Image ID	Acquire Time	Channels	Resolution	Intensities	Quality	Analysis	Image Name	Comment
1	0023124_02	May 6, 2022 2:57:13 PM	700 800	169um	Auto Auto	medium	Manual	Figure 3 B	Antigens mouse

Image Display Values

Channel	Color	Minimum	Maximum	K
700	Gray Scale (Black on White)	0.142	4.91	0

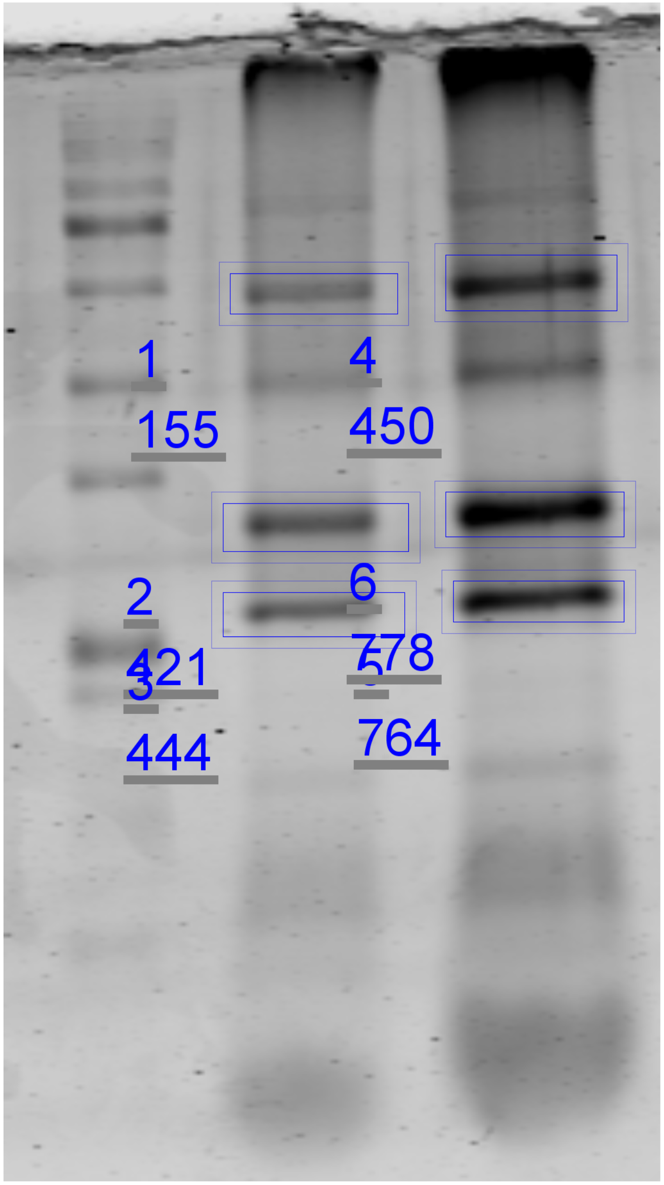

Image ID: 0026017_09
Acquire Time: May 30, 2023 2:43:28 PM

Acquisition Information (continued)

#	Image Modifications
1	Flip Right to Left Image ID: 0023124_01

Image ID: 0023729_02
Acquire Time: Aug 8, 2022 4:25:32 PM

Acquisition Information

#	Image ID	Acquire Time	Channels	Resolution	Intensities	Quality	Analysis	Image Name
1	0023704_02	Aug 5, 2022 3:47:21 PM	700 800	169um	Auto Auto	medium	Manual	FIgure S1 A

Image Display Values

Channel	Color	Minimum	Maximum	K
700	Gray Scale (Black on White)	0.406	10.2	0

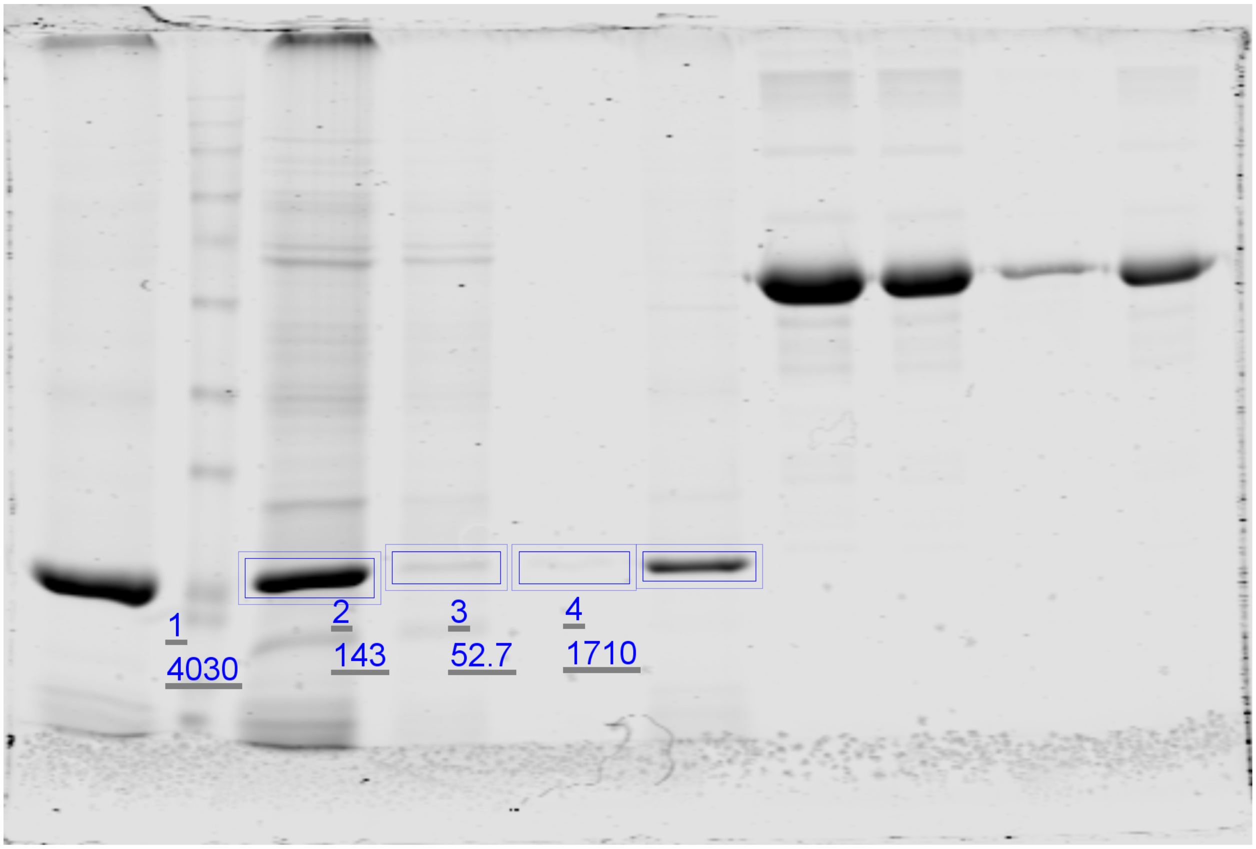

Image ID: 0023729_02
Acquire Time: Aug 8, 2022 4:25:32 PM

Acquisition Information (continued)

#	Comment	Image Modifications
1	purificacion invasina 05_08_2022	Flip Top to Bottom Image ID: 0023704_01

Image ID: 0023729_02
Acquire Time: Aug 8, 2022 4:25:32 PM

Acquisition Information

#	Image ID	Acquire Time	Channels	Resolution	Intensities	Quality	Analysis	Image Name
1	0023729_02	Aug 8, 2022 4:25:32 PM	700 800	169um	Auto Auto	medium	Manual	Figure S1 B

Image Display Values

Channel	Color	Minimum	Maximum	K
700	Gray Scale (Black on White)	0.444	7.25	0

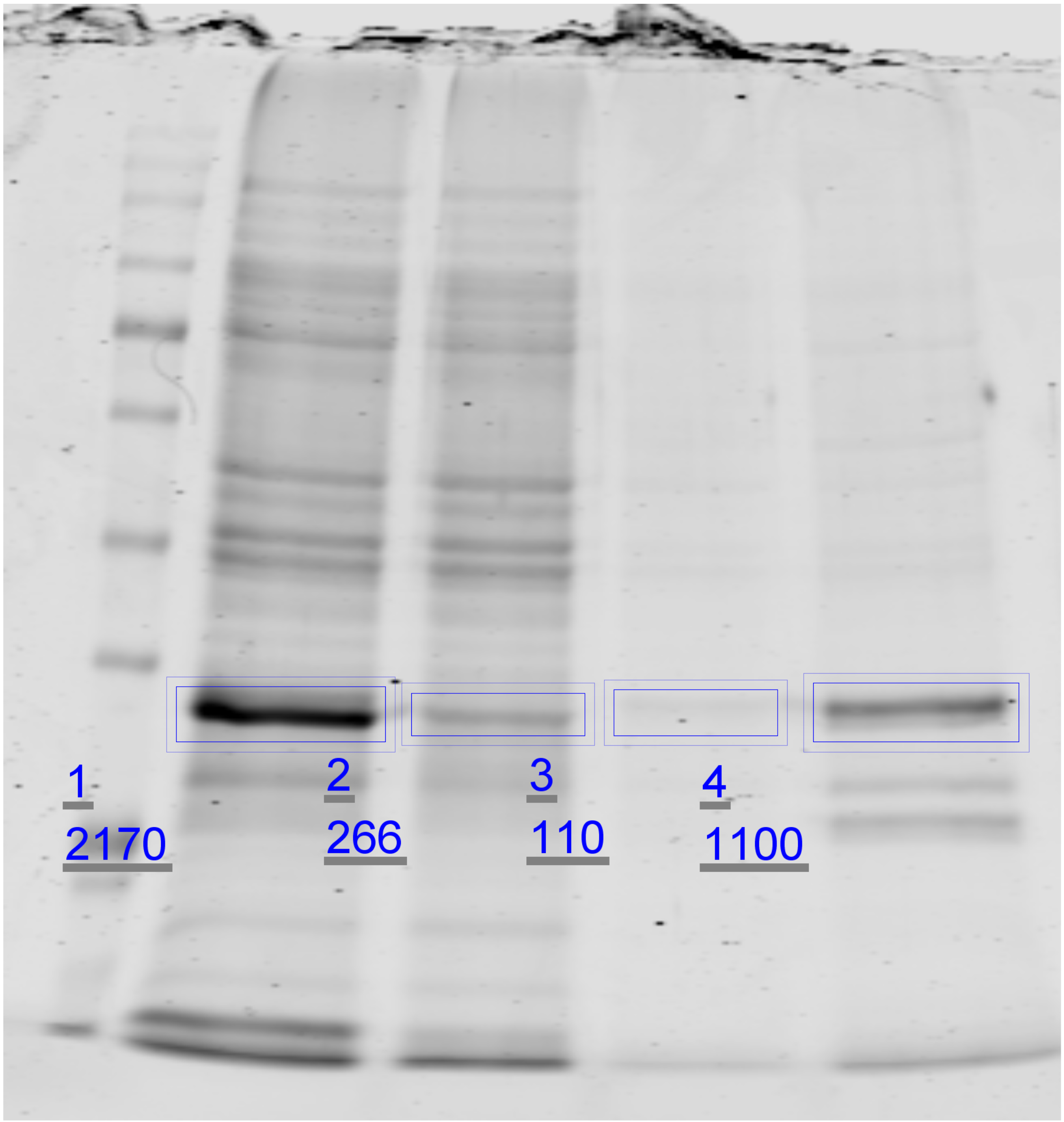

Image ID: 0023729_02
Acquire Time: Aug 8, 2022 4:25:32 PM

Acquisition Information (continued)

#	Comment	Image Modifications
1	OMP1 purification SDS PAGE	Rotate 180 Image ID: 0023729_01

Image ID: 0022926_02
Acquire Time: Apr 12, 2022 6:52:46 PM

Acquisition Information

#	Image ID	Acquire Time	Channels	Resolution	Intensities	Quality	Analysis	Image Name
1	0022926_02	Apr 12, 2022 6:52:46 PM	700 800	169um	Auto Auto	medium	Manual	Figure S1 C

Image Display Values

Channel	Color	Minimum	Maximum	K
700	Gray Scale (Black on White)	1.67	7.17	0

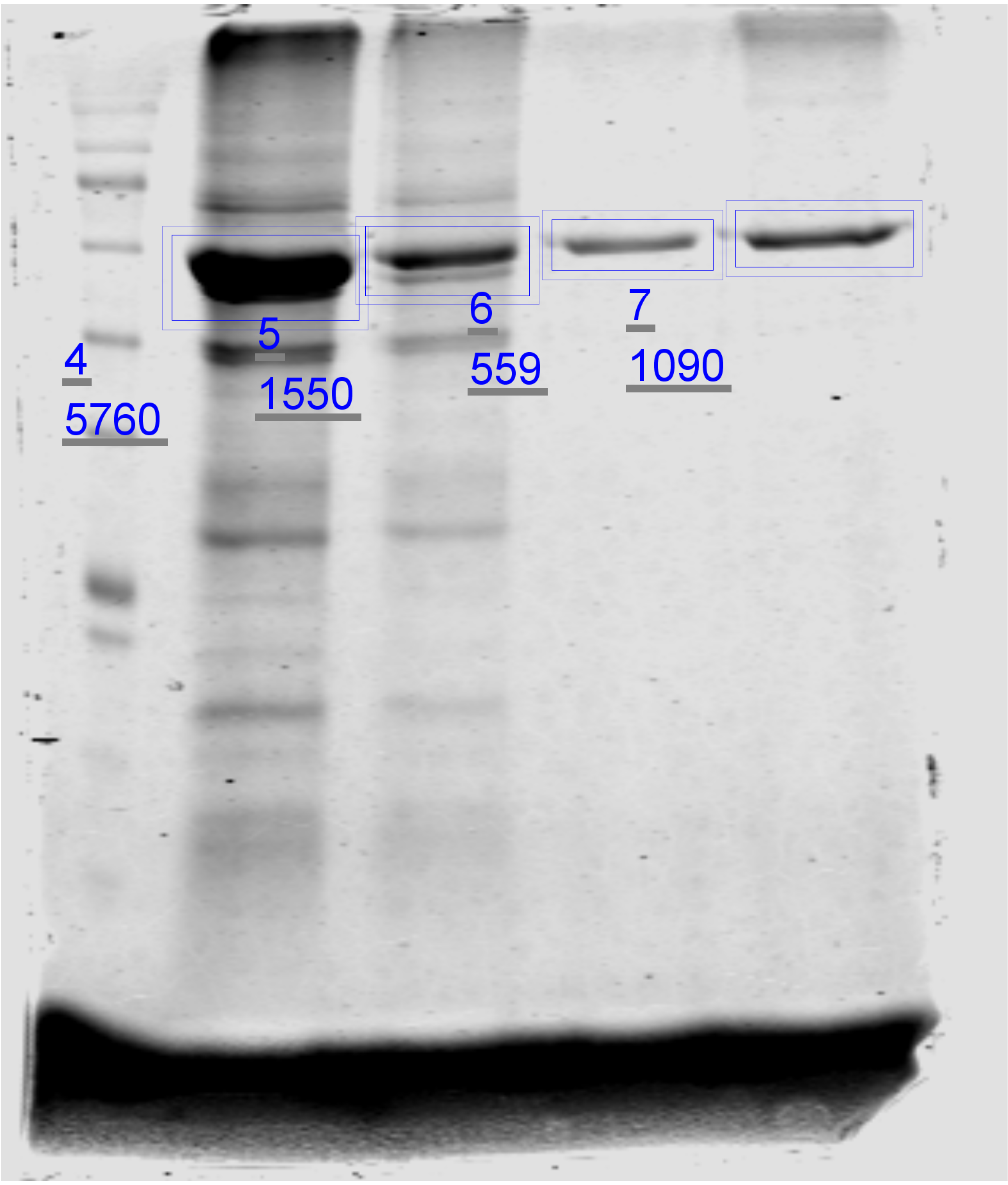

Image ID: 0022926_02
Acquire Time: Apr 12, 2022 6:52:46 PM

Acquisition Information (continued)

#	Comment	Image Modifications
1	Purificacion OMP 2 AKTA _ Inicial _ No unida_ Lavadao 10 mM _ Elucion 100 mM	Flip Top to Bottom Image ID: 0022926_01

Image ID: 0025903_03
Acquire Time: May 23, 2023 7:58:52 PM

Acquisition Information

#	Image ID	Acquire Time	Channels	Resolution	Intensities	Quality	Analysis	Image Name	Comment
1	0025903_03	May 23, 2023 7:58:52 PM	700 800	169um	Auto Auto	medium	Manual	Figure S2 A	Control

Image Display Values

Channel	Color	Minimum	Maximum	K
700	Red	3.83	26.0	0.04
800	Green	0.0560	53.3	0

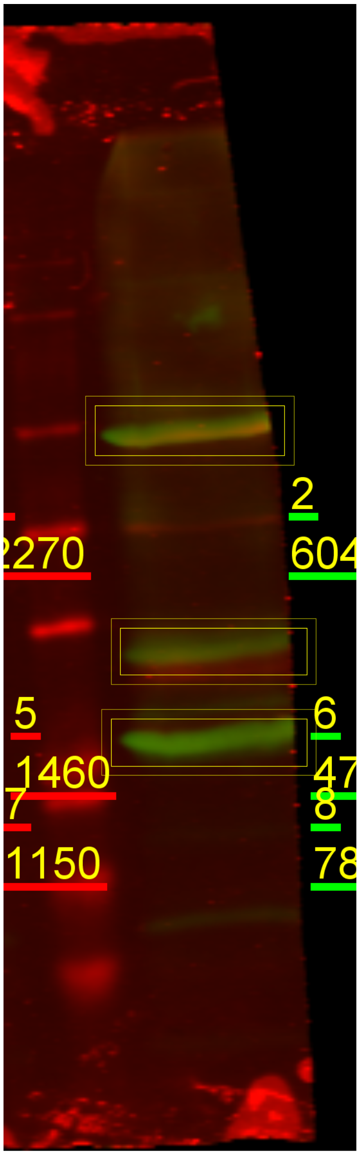

Image ID: 0025903_03
Acquire Time: May 23, 2023 7:58:52 PM

Acquisition Information (continued)

#	Image Modifications
1	Flip Top to Bottom Image ID: 0025903_01

Image ID: 0025968_01
Acquire Time: May 29, 2023 10:27:43 AM

Acquisition Information

#	Image ID	Acquire Time	Channels	Resolution	Intensities	Quality	Analysis	Image Name	Comment
1	0025968_01	May 29, 2023 10:27:43 AM	700 800	169um	Auto Auto	medium	Manual	Figure S2 B	Recombinant vaccine

Image Display Values

Channel	Color	Minimum	Maximum	K
700	Red	2.72	195	0.7
800	Green	2.67	18.8	0.2

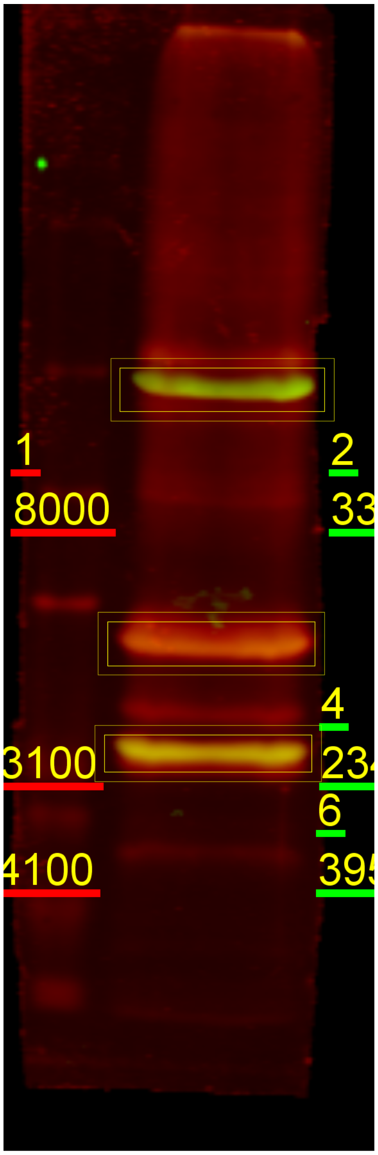

Image ID: 0025968_01
Acquire Time: May 29, 2023 10:27:43 AM

Acquisition Information (continued)

#	Image Modifications
1	

Image ID: 0025968_01
Acquire Time: May 29, 2023 10:27:43 AM

Acquisition Information

#	Image ID	Acquire Time	Channels	Resolution	Intensities	Quality	Analysis	Image Name	Comment
1	0025972_02	May 29, 2023 10:44:23 AM	700 800	169um	Auto Auto	medium	Manual	Figure S2 C	Commercial vaccine

Image Display Values

Channel	Color	Minimum	Maximum	K
700	Red	9.20	30.7	0
800	Green	14.2	54.4	0

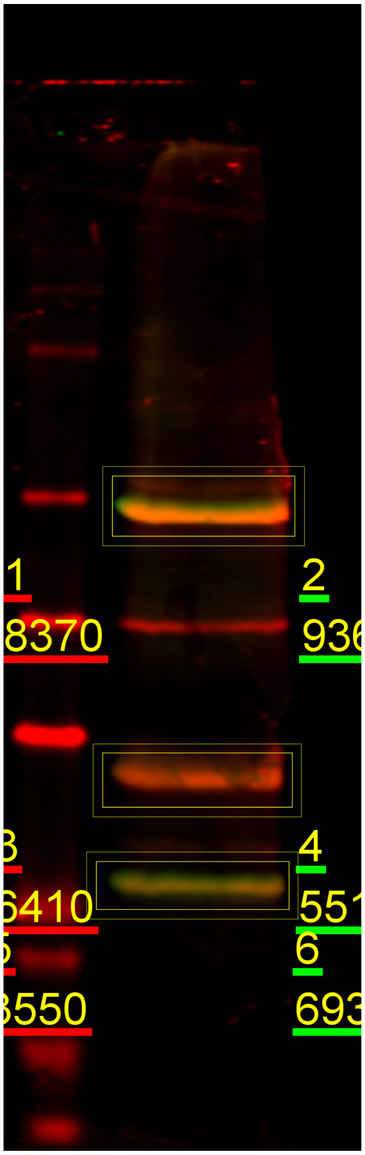

Image ID: 0025968_01
Acquire Time: May 29, 2023 10:27:43 AM

Acquisition Information (continued)

#	Image Modifications
1	Flip Top to Bottom Image ID: 0025972_01
